# PACAP and migraine headache: immunomodulation of neural circuits in autonomic ganglia and brain parenchyma

**DOI:** 10.1186/s10194-018-0850-6

**Published:** 2018-03-13

**Authors:** James A. Waschek, Serapio M. Baca, Simon Akerman

**Affiliations:** 1 Department of Psychiatry and Biobehavioral Sciences, Semel Institute for Neuroscience and Human Behavior, David Geffen School of Medicine, University of California Los Angeles, Los Angeles, CA 90095 USA; 2 Department of Pharmacy and Pharmaceutical Sciences, University of Colorado, Anschutz Medical Campus, Aurora, CO 80045 USA; 3 Department of Oral and Maxillofacial Pathology, Radiology and Medicine, New York University College of Dentistry, New York, NY 10010 USA; 4 Department of Neural and Pain Sciences, University of Maryland Baltimore, Maryland, Baltimore, MD 21201 USA

**Keywords:** PACAP, VIP, Migraine, Headache, Inflammation

## Abstract

The discovery that intravenous (IV) infusions of the neuropeptide PACAP-38 (pituitary adenylyl cyclase activating peptide-38) induced delayed migraine-like headaches in a large majority of migraine patients has resulted in considerable excitement in headache research. In addition to suggesting potential therapeutic targets for migraine, the finding provides an opportunity to better understand the pathological events from early events (aura) to the headache itself. Although PACAP-38 and the closely related peptide VIP (vasoactive intestinal peptide) are well-known as vasoactive molecules, the dilation of cranial blood vessels *per se* is no longer felt to underlie migraine headaches. Thus, more recent research has focused on other possible PACAP-mediated mechanisms, and has raised some important questions. For example, (1) are endogenous sources of PACAP (or VIP) involved in the triggering and/or propagation of migraine headaches?; (2) which receptor subtypes are involved in migraine pathophysiology?; (3) can we identify specific anatomical circuit(s) where PACAP signaling is involved in the features of migraine? The purpose of this review is to discuss the possibility, and supportive evidence, that PACAP acts to induce migraine-like symptoms not only by directly modulating nociceptive neural circuits, but also by indirectly regulating the production of inflammatory mediators. We focus here primarily on postulated extra-dural sites because potential mechanisms of PACAP action in the dura are discussed in detail elsewhere (see X, this edition).

## Review

## Introduction

Primary headaches, such as migraine and the trigeminal autonomic cephalalgias (TACs), are highly prevalent and debilitating neurological disorders that cause significant quality of life burdens for the sufferers [49, 101]. While their pathophysiology is not fully understood, the headache component is generally considered to involve neurovascular mechanisms. Migraine pathophysiology, however, most likely starts within the brain, as suggested by premonitory symptoms [47], complementary imaging studies [92], and by the nature of typical triggers, such as stress, sleep deprivation, skipping meals, and even over-sleeping [70]. Two mechanistic steps regarding the pathophysiology of the headache in both migraine and TACs are generally now accepted. First, headache pain is mediated by activation and sensitization of the trigeminovascular pain pathway [57, 108, 109], i.e., the sensory nerves that innervate the cranial vasculature, particularly the intracranial dural vasculature and large cerebral arteries, as well as extracranial blood vessels such as the temporal artery. Second, the experience of headache likely involves the release of sensory neuropeptides, including calcitonin gene-related peptide (CGRP), vasoactive intestinal peptide (VIP) and also pituitary adenylate cyclase-activating peptide (PACAP) [52–56, 154]. However, the mechanisms of brain dysfunction that lead to these primary headache disorders, and more specifically to activation of the trigeminovascular pain pathway, remain largely unknown.

### Inflammation and primary headaches

The idea that inflammatory processes are involved in headache was discussed in the literature at least as early as the 1950s, and was summarized with respect to migraine by Moskowitz in 1984 [100]. The term “neurogenic inflammation” (NI) has been applied in migraine research and was originally defined as a physiological mechanism resulting in dilation, plasma protein extravasation (PPE)-evoked oedema, mast cell degranulation, as well as other manifestations mediated by the release of neuropeptides from peripheral and central afferent nerve terminals. Support for this mechanism in migraine is predominantly based on animal studies, and historically revolves around mainly dural-mediated mechanisms. This posits that a sterile inflammatory state is induced by neuronal activity in or around the dural meninges, and underlies the sustained activation of peripheral meningeal nociceptors. This ultimately leads to sensitization of trigeminal primary afferents, as well as second-order central trigeminovascular neurons [24, 113]. This idea is clearly supported by animal experiments in which application of a mixture of inflammatory mediators to the dura mater leads to peripheral and central trigeminovascular sensitization [25, 131]. An important role for neuro-inflammation is also indirectly supported by findings of increased levels of several pro-inflammatory mediators in the cephalic venous outflow during spontaneous migraine [120], and by the efficacy of non-steroidal anti-inflammatory drugs in acute migraine treatment [51, 58, 112], as well as their success in reversing peripheral and central sensitization in animal models [77, 78].

The presence of pro-inflammatory mechanisms and release of mediators within and around the dural micro-environment, and its subsequent effects on trigeminovascular neurons, are very likely to cause cephalic pain similar to migraine headache. That said, when the individual component parts of NI are assessed as potential contributors to eventual trigeminovascular activation and primary headaches, their contributions appear less clear. For example, the vasodilation of dural blood vessels was long thought to be responsible for throbbing head pain, particularly in migraine. However, more recent studies demonstrate that meningeal vessels do not necessarily dilate during spontaneous [10] or experimentally-triggered migraine attacks [123]; intracranial vessels show only a slight dilation that is unaffected by sumatriptan treatment [10]. Also, the throbbing and pulsatile nature of migraine headache is not coupled to the frequency of arterial pulsations [97, 98], but rather appear coupled to endogenous brain oscillations related to alpha power [98]. Finally, although several vasoactive drugs cause cranial arterial vasodilation and subsequently trigger migraine, including nitroglycerin [76], CGRP [14, 85], and PACAP [11, 124], VIP [11, 114] does not trigger a delayed migraine headache in patients. Indeed, in a recent preclinical study it was found that both VIP and PACAP similarly cause short-lived (1–5 min) vasodilation of meningeal arteries, yet only PACAP was able to trigger a delayed, by 90 min, activation and sensitization of central trigeminovascular neurons [5].

Inhibition of dural PPE was once a major platform used in screening the efficacy of drugs such as sumatriptan for the treatment of migraine [26, 27, 94]. Dural PPE can be mediated by trigeminal ganglion stimulation, or the systemic application of various chemical mediators, including substance P, capsaicin and neurokinin A [93]. However, drugs known to trigger migraine, including CGRP and prostaglandin E2 (PGE2) [12] do not cause dural PPE, and there is no evidence of release of substance P in cephalic venous outflow during spontaneous migraine [56], in contrast to CGRP and PGE2. Furthermore, several drug classes screened as potential acute migraine therapeutics, defined by their ability to inhibit dural PPE, including specific extravasation inhibitors (i.e., conformationally restricted analogues of triptans) [42, 118], neurokinin 1 receptor antagonists [60, 61], and inducible nitric oxide synthase inhibitors [72], were all ineffective clinically as either acute or preventive treatments.

Despite these negative outcomes arising from using PPE as a surrogate for inflammation in migraine, dural mast cell degranulation clearly represents a relevant mechanism that might lead to activation and sensitization of the trigeminovascular pain pathway. Upon activation, mast cells are known to degranulate and release a host of inflammatory mediators. Exogenous migraine triggers, CGRP, NTG and PACAP, can cause dural mast cell degranulation [18, 110, 116], and subsequent release of inflammatory mediators. Furthermore, specific dural mast cell degranulation produces a long-lasting sensitization of trigeminal primary afferent neurons [88, 156], suggesting this neuro-inflammatory mechanism may be directly involved in mediating the underlying neurophysiological changes that results in primary headache, particularly in migraine.

The important role of neuropeptides, such as CGRP and PACAP, in the pathophysiology of migraine seems undeniable, and it is clear that this may involve elements of neurogenic dural inflammation. However, beyond the dura mater and the trigeminovascular pain pathway (topics of other reviews within this special issue, and also reviewed recently [133]), PACAP-regulated neuro-inflammatory mechanisms within deeper brain structures may be involved in mediating these dural changes, such as in the brainstem, cortex, or autonomic projections, which lead to trigeminovascular activation. Furthermore, other neuro-inflammatory mechanisms within these structures may directly mediate activation of the trigeminovascular pain pathway without impacting the dural vasculature. The aim of the remainder of this review is to focus on the specific role of PACAP in neurogenic inflammation. Furthermore, we will focus on how PACAP-mediated neurogenic inflammation in deeper brain structures may contribute to the underlying nociceptive neurophysiology of primary headaches, especially in migraine.

### PACAP signaling

PACAP-38 is a 38 amino acid long neuropeptide originally isolated in 1989 in a search for novel factors produced in the hypothalamus that could stimulate cAMP production in pituitary cells. Upon sequencing, PACAP (gene name Adcyap1) was found to be 68% homologous to the neuropeptide vasoactive intestinal peptide (gene name VIP). A 27 amino acid long C-terminal truncated form, PACAP-27, is internally cleaved from PACAP-38, and is generally produced in lower but significant concentrations in many of the same sites as PACAP-38, and has similar biological activities and receptor binding affinities. The two PACAP species are exceptionally-well conserved across evolution. For example, human PACAP-27 is 97% identical to that of amphibians [75] and 90% to that of hydra and other cnidarians [28]. PACAP (both forms) bind to three different receptors: PAC1, which specifically interacts with PACAP, and VPAC1 and VPAC2, which bind PACAP and VIP with approximately equal affinity [67]. After its discovery, PACAP was found to function in vertebrates as a neurotransmitter/neuromodulator in many processes in the central and peripheral nervous systems, such as in the control of circadian rhythms, learning and memory, and reproduction, and has been implicated in multiple forms of stress, including metabolic, hemodynamic, and emotional stress (reviewed in [32, 35, 44, 65, 68, 102, 115, 125, 132, 138, 145, 149]). In addition, considerable evidence indicates that PACAP functions broadly in brain development to regulate cell proliferation, maturation and survival [9, 44, 106, 129, 146–148], and to provide neuroprotection and promote repair after injury (reviewed in [32, 115, 125]). As discussed below, considerable evidence indicates that another important function of VIP and PACAP is to modulate the activity of inflammatory cells. The development and clinical testing of PACAP and/or VIP antagonists for migraine will need to carefully take into account how a widespread and/or uncontrolled blockade of these receptors might affect the known homeostatic activities of these endogenously-expressed peptides.

### Which receptor subtype/s mediate the migraine-inducing action of PACAP?

**Fig. 1:**
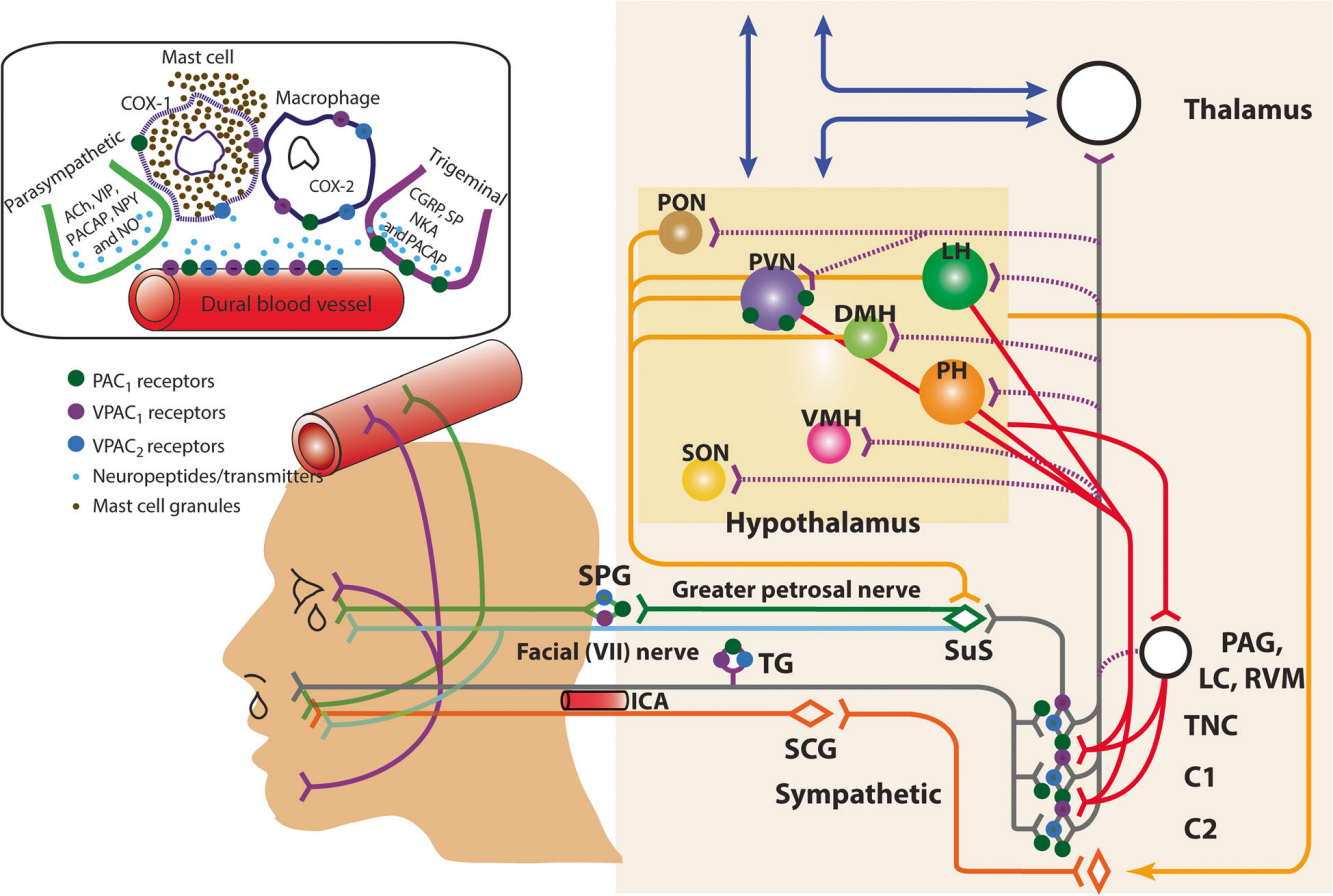
Anatomy and PACAP-mediated cranial trigeminal-autonomic mechanisms mediating dural-trigeminovascular activation. Cranial autonomic symptoms are thought to be mediated, in part, by activation of the trigeminal-autonomic reflex; a reflex connection from the trigeminal nucleus caudalis (TNC; grey neuron), via the superior salivatory nucleus (SuS; green diamond), which provides an autonomic parasympathetic projection to the cranial vasculature. This is predominantly through the greater petrosal nerve (green neuron) and its relay with the sphenopalatine ganglion (SPG), but also via the facial (VIIth cranial) nerve (sky blue neuron). Descending projections from hypothalamic nuclei (red and yellow neurons) including the posterior (PH), paraventricular (PVN), lateral (LH), dorsomedial (DMH) and pre-optic hypothalamic nuclei (PON), to the TCC (red projections) and SuS (yellow projections) neurons, are thought to modulate and control both trigeminovascular nociceptive transmission (purple network of neurons) and parasympathetic (green) autonomic projections to the cranial vasculature that result indirectly or directly, respectively, in cranial autonomic symptoms ipsilateral to head pain. Cranial autonomic symptoms, and activation of the cranial autonomic projection, are thought to modulate or even trigger activation of dural neuro-inflammatory mechanisms, which mediate dural trigeminovascular activation resulting in headache in primary headache. Activation of preganglionic SuS neurons stimulates the release of various neurotransmitter (light blue dots), including PACAP-38, VIP, neuropeptide (NPY), acetylcholine (ACh), and nitric oxide (NO) from nerve terminals of postganglionic parasympathetic neurons in the SPG. Their release is thought to mediate meningeal vasodilation and dural mast cell degranulation (brown dots), the production of COX-1 from mast cells and COX-2 from macrophage, causing the local release of inflammatory mediators, together capable of activating pial and dural branches of the trigeminal nerve. The presence of mRNA and/or protein for VPAC1/2 and PAC1 receptors in human/rat middle meningeal arteries, trigeminal ganglia and trigeminal nucleus caudalis (TNC), and sphenopalatine ganglia (SPG), mast cells and macrophages, suggest PACAP signaling mechanisms are involved in mediating cranial autonomic symptoms, but also in mediating dural neuro-inflammatory mechanisms that contribute to dural trigeminovascular activation. CGRP, calcitonin gene-related peptide; SP, substance P; NKA, neurokinin A; VMH, ventromedial hypothalamus; SON, supra-optic nerve, TG, trigeminal ganglion, SCG, superior cervical ganglion, PAG, periaqueductal gray; LC, locus coeruleus; RVM, rostral ventromedial medulla

**Fig. 2:**
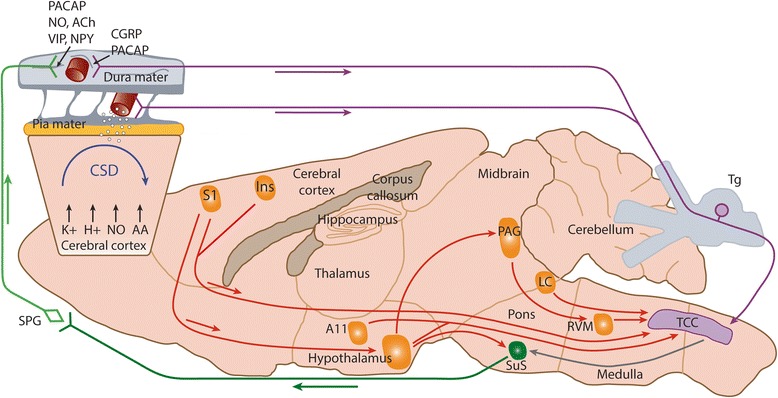
Descending brainstem and cortical modulation of dural-trigeminovascular mechanisms in primary headache.  Descending projections from brainstem nuclei including the ventrolateral periaqueductal grey (vlPAG), locus coeruleus (LC) and raphe/rostral ventromedial medullary (RVM) nuclei provide modulation of noxious somatosensory dural-trigeminovascular inputs. Noxious peripheral inputs and central descending modulation are integrated within trigeminocervical (TCC) neurons, the net result of which is head pain, processed within the thalamocortical neurons. Either direct activation of preganglionic pontine superior salivatory nucleus neurons from descending hypothalamic projections, or via reflex activation of trigeminal-autonomic relay, leads to activation of the cranial parasympathetic projection, which can trigger or exacerbate the dural neuro-inflammatory cascade. Activation of cortical neurons within the somatosensory (S1) and insulae cortices, via cortical spreading depression, and direct descending projections, can also facilitate or inhibit dural-trigeminovascular responses within the TCC. In addition, CSD is thought to directly mediate a neuro-inflammatory response within the dural micro-environment to activate trigeminal primary afferent neurons that innervate the dural vasculature. CSD is proposed to activate headache by initiating a complex cascade where neurons open pannexin1 channels that activate caspase-1 and the release of pro-inflammatories such as HMGB1 and IL-1B. Following pro-IM release, NF-KB translates to the nucleus to induce COX2 and iNOS expression in astrocytes. The activated astrocytes release cytokines, prosanoids, and NO to the subarachnoid space and produce sustained activation of trigeminal nerve fibers. Trigeminal fiber collaterals produce a sterile dural inflammation that lead to mast cell degranulation and the trigeminoparasympathetic reflex causes a late and sustained medial meningeal artery dilation (see Fig. S6 in Katata et al. [79] for more details.). In the CSD rodent model, facial grimace assessments suggest the final step in the parenchymal signaling cascade outlined above produces headache

As discussed, the perception of migraine headache is believed to critically involve enhanced sensitivity of trigeminal pain circuits. PACAP receptors are expressed in trigeminal, sympathetic, and parasympathetic nerve terminals in the dura, but also in neurons at several levels of the migraine circuity within the CNS **(**Figs. 1 and 2). Potential access to intravenously-administered PACAP to the CNS is discussed below. In addition to their presence on neurons, receptors for PACAP (most prominently, VPAC1, and VPAC2, but also PAC1) are expressed on nearly all cells of the immune system, including the resident macrophages of the brain, microglia. Moreover, all three receptors are expressed on astrocytes, which under some conditions are known to produce inflammatory mediators.

Pharmacological studies suggest PACAP receptor subtypes might mediate the action of systemically administered PACAP. Intravenous infusions of PACAP-38, but not VIP, reliably and repeatedly induce delayed migraine-like headaches. Given that the PAC1 receptors are at least two orders of magnitude more selective for PACAP than VPAC1 and VPAC2 receptors, it is tempting to conclude that PACAP acts in this situation via action on PAC1 receptors. However, such a conclusion is premature. A trivial, but as yet untested mechanism is that PACAP may be more stable in the blood than VIP. This is supported by pharmacokinetic findings which indicate a half-life of two minutes for VIP [69] and 5–10 min for PACAP [89]. Thus, infusion of PACAP might be expected to produce considerably higher steady state blood concentrations than equimolar infusions of VIP. Another possibility is that PACAP has better access to the relevant sites of action, for example by way of better penetration of the blood-brain-barrier. Moreover, putative PACAP and VIP receptors on mast cells and neutrophils do not at all correspond to known receptors for these peptides, showing activation in response to both receptor agonists and antagonists [18]. Finally, recent research shows that PACAP can penetrate cells raising the possibility that PACAP might act on an as yet unknown intracellular receptor [40]. Thus, further investigation is needed to identify the receptor or receptors that mediate the ability of intravenous administration of PACAP to trigger migraine-like headaches.

### Immunomodulatory actions of PACAP and VIP in the periphery

The preponderance of literature describing the importance of PACAP and VIP on inflammation has addressed their roles in regulating innate and adaptive immune processes in the periphery (extensively reviewed in ref. [35]). In this regard, PACAP and/or VIP are expressed in autonomic neurons that innervate all major lymphoid structures, including lymph nodes, spleen, thymus, and bone marrow, and nearly all immune cell types express one or more VIP and PACAP receptor subtypes. Under some circumstances the peptides themselves appear to be expressed in the mast cells and lymphocytes, where they may function like cytokines or chemokines. One of the earliest-studied activities of these peptides on immune function was their capacity to inhibit inflammation through their action on macrophages. These cells constitutively express VPAC1 and PAC1 receptors, and when exposed to an inflammatory stimulus, express VPAC2. Early studies reported that VIP and PACAP inhibited the production of TNF-α and IL-6 in macrophage cultures in response to lipopolysaccharide (LPS). Later, these peptides were shown to increase the synthesis and release of anti-inflammatory molecules like IL-10 and the IL-1 receptor antagonist (IL-1Ra), leading to a decrease in the inflammatory response [34]. VIP and PACAP were also shown to inhibit the production of several chemokines in macrophage cultures stimulated with LPS [33]. The ability of PACAP to trigger mast cell degranulation is reviewed elsewhere in this series (Jansen-Olesen). A few studies have addressed the potential actions of these peptides on other innate immune cells including, granulocytes, natural killer (NK) cells, and NKT cells (reviewed in Delgado [35]). All of these cell types are present in the dura of rats, but further work needs to be done to clarify potential actions of PACAP and VIP on these cells.

VIP and PACAP are known to regulate T cell function as evidenced by studies of their anti-inflammatory actions in animal models of autoimmune/auto-inflammatory disease, including those modeling multiple sclerosis, rheumatoid arthritis, and inflammatory bowel disease. Adaptive immunity is not widely thought to have a role in migraine, although a few studies support this possibility (for example, [13]). In general, PACAP and VIP promote Th2 and regulatory T cell production, stability and function at the expense of Th1 and Th17 phenotypes. Readers are referred to ref. [35] for more detailed information on the important actions of these peptides in adaptive immunity.

### Access of blood-born PACAP to the brain parenchyma

With a few exceptions, peptides present in the blood cannot penetrate well into the parenchyma of most regions of the brain due to specialized tight-junction molecules that line endothelial cells in blood vessels within the CNS (the endothelial component of the blood brain barrier (BBB)). Certain structures within the brain, referred to as the circumventricular organs lack this BBB. In particular, it is thought that the area postrema, the subfornical organ, and the vascular organ of lamina terminalis can receive peptide and other blood-born signals, and transmit information to other parts of the brain. In particular, the area postrema is of interest because *in situ* hybridization shows that PAC1 receptor gene transcripts are high in this structure. Neurons in the area postrema send major efferents to the nucleus of the solitary tract (NTS) and the lateral parabrachial nucleus and the hypothalamus. Minor efferents are reported to project to several other regions such as the nucleus ambiguus, dorsal motor nucleus of the vagus, dorsal regions of the tegmental nucleus, cerebellar vermis and ventrolateral catecholaminergic column in the medulla [87, 127].

### Glial cells in the brain parenchyma: Potential roles in synaptic transmission and modulation by PACAP

Microglia are often viewed as the resident macrophages of the brain. When activated, they produce and release inflammatory molecules, including pro-inflammatory cytokines, matrix metalloproteinases, and free radicals. They also function in repair by secreting neuroprotective and regenerative factors. Several in vitro studies have demonstrated the capacity of PACAP and VIP to potently inhibit the release of inflammatory factors from microglia via action on VPAC and PAC1 receptors. This argues against a role for a PACAP/microglia interaction in migraine. On the other hand, in the uninjured brain, microglia appear to play important roles in synapse formation (in part by secreting BDNF) and in the remodeling of synapses in the processes of learning and memory. Moreover, glia-derived TNF-α was reported to be necessary and sufficient for synaptic upscaling after chronic activity blockade. Imaging studies aided with fluorescent genetic probes have shown that microglia continually extend and retract their processes [107]. Microglia actions such as these are conceivably involved in the delayed migraine-like headaches. The potential for PACAP to regulate synaptic functions via microglia has yet to be examined.

Like microglia, astrocytes are known to secrete inflammatory molecules in the settings of brain injury, neurodegeneration, and repair, and have important synaptic functions in the uninjured brain. Astrocyte processes are well known to surround synapses and express proteins that regulate synapse function, including enzymes that control glutamate metabolism, shuttle lactate and other energy substrates, regulate water movement between cells, buffer potassium ions and mediate membrane adhesion. The fact that PAC1 receptor expression is upregulated in astrocytes in several CNS injury models such as global ischemia [103], contusion spinal cord injury [141], and cortical stab injury [135], suggest that PAC1 receptors in these cells might have a role in protection and repair. PACAP was also shown to very potently increase IL-6 production in primary cultures of rat astrocytes [62]. Others have shown that PACAP induces the expression of several chemokines in cultured astrocytes including RANTES and MIP1α [22], highlighting potential pro-inflammatory actions of PACAP on astrocytes. Perhaps more relevant to astrocyte-mediated effects of PACAP on synaptic activity, in vitro studies indicate that PACAP/PAC1 signaling increases glutamate uptake via induced expression of GLT-1/EAAT2, GLAST/EAAT1 and increases glutamine synthase (GS) [121].

### Specific sites within the CNS where PACAP might trigger migraine pathophysiology

As discussed, neurogenic inflammation in relation to primary headaches is most commonly associated with dural mechanisms. However, several studies suggest neuro-inflammation may also occur in other regions of the brain. Application of the migraine trigger, NTG, not only promotes dural changes, but also induces neuronal activation in brainstem and higher pain processing nuclei, such as the periaqueductal grey and hypothalamic nuclei [140]. Cortical spreading depression (CSD), believed to be the neurophysiological correlate of aura in migraine [86], may induce a cortical neuro-inflammatory cascade that leads to activation and sensitization of the trigeminovascular pain pathway. The remainder of this review will focus on the role of PACAP in neurogenic inflammation, and how this might relate to mediating primary headache mechanisms.

### PACAP and spinal nociception

Spinal nociception is likely to have some similar mechanistic underpinnings as trigeminal nociception, and is in some respects more amenable to study. Considerable work has examined the roles of PACAP and VIP in spinal nociception (reviewed in [38, 136]). Like in the trigeminal ganglia, PACAP is expressed in a subpopulation of neurons in dorsal root ganglia. PACAP immunoreactive fibers are highly abundant in the superficial laminae of the dorsal horn, where all three receptors appear to be expressed. Iontophoretic application of PACAP-38 was shown to exert excitatory actions on dorsal horn neurons [39]. Pharmacological studies in various acute and chronic pain models generally support a pro-nociceptive action of PACAP-38, and mice deficient in PACAP and PAC1 receptors fail to develop hypersensitivity to nociceptive stimuli in neuropathic and inflammatory pain paradigms. The involvement of PACAP receptors on glial cells on sensitizing pain pathways in the spinal cord is currently unknown, although one study showed that intrathecal PACAP administration resulted in long-lasting hind paw allodynia and sustained activation of astrocytes [153]. In the target tissues, intradermal injection of PACAP was found to induce localized pain and edema in humans and pain-like behavior in rodents reminiscent of neurogenic inflammation. The number of CD31+ vessel cross sections in organotypic human skin cultures was found to be increased in response to PACAP-38 application without evidence of mast cell degranulation [126], whereas PACAP-38-induced dermal edema was abolished in c-kit mutant mice that are deficient in mast cells [122] . Thus, the role of mast cells in the neurovascular response is unclear.

### PACAP and the parasympathetic pathway

Cranial autonomic symptoms, such as conjunctival injection, lacrimation, nasal congestion, rhinorrhea, eyelid edema and forehead/facial sweating, can be hugely debilitating, and they are a prominent and defining feature of TACs, such as cluster headache [29, 71, 96]. They are also prevalent in up to 50% of migraine patients [16, 17, 83]. Their presence is thought to exacerbate the general migrainous phenotype [16, 17]. These data suggest, first, a likely overlap of pathophysiology between migraine and TACs as they relate to cranial autonomic symptoms; and, second, that the presence of these symptoms may be relevant to the pathophysiology in these primary headache disorders, and may contribute, or even trigger trigeminovascular activation and sensitization.

Cranial autonomic symptoms are thought to be mediated, in part, by activation of the trigeminal autonomic reflex, and the parasympathetic autonomic projection to the cranial vasculature [50, 63]. A reflex connection from the TNC to the preganglionic parasympathetic neurons in the pontine superior salivatory nucleus (SuS), is thought to connect these two important somatosensory and autonomic pathways (see Fig. 1). The SuS is the origin of cells of the parasympathetic vasodilator pathway, and it projects to the cranial vasculature, including the dura mater and the lacrimal gland, predominantly via the greater petrosal nerve (green nerve), and its synapse with the sphenopalatine ganglion (SPG), and the VIIth (facial) nerve (light blue nerve) [130]. Both the TNC and SuS receive descending projections from hypothalamic and brainstem nuclei including, the lateral and paraventricular hypothalamic nuclei [59, 73, 74, 117, 130]. This might illustrate a mechanism in triggering both headache and cranial autonomic symptoms in these primary headaches.

Activation of the cranial parasympathetic vasodilator pathway might also influence both central trigeminovascular neurons, and the dural microenvironment, potentially by evoking neuro-inflammatory mechanisms. Activation of this neurovascular pathway with its cranial autonomic symptoms may also affect the neurophysiology related to head pain in migraine and cluster headache. The dural blood vessels are richly innervated by parasympathetic nerve fibers [134] and activation of this pathway causes the release of acetylcholine [128], VIP and NO, from dural vascular terminals of post-ganglionic sphenopalatine neurons, also containing PACAP. Activation can lead to dilation of intracranial vessels, plasma protein extravasation and local dural release of inflammatory mediators [23, 24], which may lead to a cascade of events that ultimately produces activation of dural-trigeminovascular neurons (see Figs. 1 and 2). Experimentally, electrical stimulation of preganglionic SuS neurons produces neuronal action potentials in the TCC via two separate neural pathways. First, via retrograde activation of the trigeminal autonomic reflex, within the brainstem. Second, however, by activation of the parasympathetic outflow to the cranial vasculature, which indirectly activates trigeminal afferents from the dura mater to the TCC, as well as producing cranial autonomic symptoms [7, 8]. These latter responses are attenuated by the specific SPG blocker, as well as by oxygen treatment. Interestingly, this is not accompanied by dilation of meningeal blood vessels [7]. These data are somewhat paralleled by clinical studies demonstrating that SPG blockade provides partial or complete relief of migraine pain [152]. Finally, activation of primary afferent dural trigeminal neurons appear to depend to some extent on SPG activity [20]. Together, these more likely maintaining, a state of trigeminovascular activation and sensitization. This may be mediated via activation of the trigeminal-autonomic reflex (most likely in maintaining the active trigeminovascular state), descending control of trigeminal somatosensory and SuS-autonomic via hypothalamic and brainstem nuclei, or a combination of both, which exacerbate the responses of either, via the release of vasoactive sensory neuropeptides, such as PACAP and VIP.

Despite the overwhelming data suggesting that cranial parasympathetic activation may be important in the modulation of trigeminovascular mechanisms related to headache, one study suggests the contrary. In preliminary studies it was observed that low frequency stimulation (LFS) of SPG seemed to trigger cluster headache in patients. These patients had surgically implanted SPG stimulator devices that used high frequency stimulation for relief of cluster headache. In a double-blind randomized sham-controlled trial, LFS was no different from ‘sham’ in inducing cluster headache, but was significantly more likely to produce cranial autonomic symptoms compared to ‘sham’ [63]. In the immediate phase LFS caused cluster headache in 35% (7/20) patients, whereas ‘sham’ caused cluster in 25% (5/20) patients. Cranial autonomic symptoms were present in 80% of LFS and 45% of sham patients. The data suggest that in a rarer headache disorder, compared to migraine, cranial parasympathetic activation cannot trigger a cluster headache attack. There is an important caveat to interpreting this data. ‘Sham’ stimulation triggered a delayed cluster headache attack (> 1–24 h) in 75% (15/20 patients) compared to 70% (14/20 patients) in the LFS group. The same numbers were found for the development of cranial autonomic symptoms. With such a high rate of response to ‘sham’ it is impossible to dissect an effect of LFS in this delayed phase. In addition, from these data, it is not possible to determine whether cranial parasympathetic activation influences the maintenance of head pain, or has a role in more common primary headache disorders, such as migraine.

PACAP-38 and VIP are both described as parasympathetic vasodilator peptides, and they are linked to cranial autonomic symptoms in migraine and TACs. There are increased levels of PACAP and VIP in the extracranial vasculature during both spontaneous migraine [56, 142, 154] and cluster headache [53, 143], although VIP levels during severe migraine are only increased when accompanied by cranial autonomic symptoms. Furthermore, both cause cranial autonomic symptoms [11, 114, 124] when given exogenously in patients, which is suggestive of activation of the parasympathetic autonomic fibers that project to the cranial vasculature. In support of the important role of PACAP and VIP in the cranial parasympathetic pathway there is evidence of both peptides in human SPG localized in neurons, and often co-localised with nitric oxide synthase [31]. There is also evidence of VPAC1, VPAC2 and PAC1 receptor expression in both human and rat SPG, but not co-localised with the PACAP and VIP [31]. There is also preliminary data suggesting that VPAC1 and PAC1 receptor antagonists are able to inhibit both cranial autonomic and trigeminocervical neuronal responses after stimulation of the SuS [4]. Together these data suggest that the cranial parasympathetic projection is ideally placed to mediate dural neuro-inflammatory mechanisms that contribute to trigeminovascular activation in primary headaches. Further, that PACAP-mediated signaling is the most likely pharmacological class involved in this pathway, and a potential target loci and pharmacology for therapeutic intervention.

### PACAP, stress, and the sympathetic nervous system.

Stress is a major trigger of migraine, suggesting that stress centers in the brain and the sympathetic nervous system play a role in migraine. PACAP is well known as a master regulator of the stress response, acting with the CNS and peripheral nervous system to increase sympathetic activity (reviewed in [46, 68, 119, 149]). In this regard, PACAP/PAC1 signaling is critically required in the hypothalamus to mediate the induction of the hypothalamic-pituitary adrenal axis and likely plays a role in other areas of the CNS that mediate responses to emotional and other types of stress, such as the amygdala, bed nucleus of the stria terminalis, and locus coeruleus (reviewed in [64]). PACAP could potentially trigger migraine by modulating neurotransmission in the brain areas involved in stress by direct action on neurons, or by triggering astroglial and microglial neuro-inflammatory responses. PACAP is also expressed in the acetylcholine-expressing preganglionic neurons in the spinal cord that innervate the sympathetic ganglia [19, 111], whereas PAC1 receptors are expressed in the postganglionic sympathetic neurons [21, 48]. PACAP-induced sympathetic activity in dural blood vessels could thus trigger migraine in susceptible individuals.

### PACAP and the brainstem

The role of brainstem nuclei in the pathophysiology of primary headaches, particularly migraine, has been extensively reviewed [6, 24, 57]. There is activation within brainstem nuclei, likely periaqueductal grey (PAG), locus coeruleus (LC) and raphe nuclei [1–3, 15, 92, 151], in addition to hypothalamic activation [36, 92], during the premonitory and headache phases that appear specific to a migraine attack. Central trigeminovascular neurons are under the control of pain modulatory circuits in the brainstem. This is clearly demonstrated in preclinical studies in rodents that show that descending projections from PAG, LC, raphe and rostral ventromedial medullary (RVM) neurons [43, 80–82, 84, 90, 91] are able to modulate noxious and non-noxious intracranial-dural somatosensory inputs within central trigeminovascular neurons (Fig. 2). Whether these brainstem nuclei are involved in triggering a migraine attack, or mediating changes within central trigeminovascular neurons that results in hypersensitive responses to normal and/or noxious stimuli coming from intracranial structures, such as the dura mater, within an attack is still debated. However, central trigeminovascular neurons are known as integrative relay neurons between peripheral and central pain mechanisms. Thus, the net result of activation, of nociceptive intracranial dural structures, and altered (dysfunctional) descending modulation of central trigeminovascular neurons, in headache, is due to an altered perception of craniovascular inputs, and also a generalized increase in sensitivity of other sensory inputs, via the modulation by these same brainstem nuclei.

PACAP and its receptors are ideally positioned to play an important role in these processes. Aside from exogenous PACAP triggering migraine attacks in migraineurs, PACAP is released endogenously during a migraine attack [142, 154]. Indeed, even during exogenously-triggered migraine with PACAP-38, levels within the cranial vasculature are higher than would be anticipated at time of sampling, suggesting that these headaches are also mediated by endogenous release of PACAP [11]. Overall this suggests PACAP-38 within the brainstem may have a physiological role to play in the pathophysiology of migraine. In support of this, immunoreactivity to PACAP-38 is present in approximately 40% of LC neuronal cells, and a smaller population within the PAG [137]. There is also evidence of receptor binding specific to PACAP in the LC, PAG and also dorsal raphe nuclei, which is indicative of PAC1 binding [95]. Studies into the role of PACAP-mediated mechanisms within the brainstem in primary headache pathophysiology are in their infancy compared to other neuropeptides, such as CGRP. However, it is possible that manipulation of PACAP containing pathways differentially modulates noxious and innocuous intra and extra-cranial somatosensory processing, similar to mechanisms described within the paraventricular hypothalamic nucleus [117].

Evidence of PACAP-mediated neuro-inflammatory mechanisms within the brainstem in migraine is limited. However, previous studies demonstrate that application of nitroglycerin, another exogenous trigger of migraine, causes increased expression of COX-2, which promotes the production of prostaglandins, including prostaglandin E2 [139]. Also, activation of microglia and astrocytes within the PAG [41, 99, 105], LC [104, 155] and raphe/RVM nuclei [37, 150] is evident during various forms of neuropathic pain, resulting in the release of inflammatory mediators. There is evidence of activation of glial cells proximate to trigeminal neurons in various animal models of migraine and craniofacial pain [30, 45, 66, 144], which might suggest there is similar activation in brainstem nuclei. At the moment, there is still much to be learned about neuroimmune responses during primary headaches, particularly in the brainstem. The established importance of PACAP and the PAC1 receptors in other immune responses suggest they are also likely to play an important role in primary headache.

### PACAP and cortical mechanisms

First identified in the 1940s, cortical spreading depression (CSD) is a profoundly disruptive neurovascular event that results in a large DC potential shift, the reversible loss of ionic homeostasis, and multi-phasic changes in blood oxygenation and blood vessel constriction-dilatation dynamics. These electrochemical and vascular events are iterated slowly across the cortex as a propagating wave that travels both across and within the cortex. CSD is present in some of the brain injury models remarked upon earlier (e.g., global ischemia, cortical stab) and is believed to be the operant mechanism that produces visual aura in migraine. CSD tends to correlate with more dire outcomes in cases of hemorrhagic stroke. The parenchymal and vascular changes observed in animal models tend to return to normalcy after about an hour. Such sustained changes in neurovascular dynamics observed in spontaneous CSD as may occur in migraine likely includes many of the same PACAP mediated changes (e.g., astrocyte activation, changes in glutamate regulation, PACAP mediated changes in neuro-inflammation) observed in brain injury models. Interestingly, astrocytes can be selectively activated optogenetically to elicit CSD and preliminary studies suggest that astrocytes may be in fact activated many seconds before the typical markers of CSD are observed. Thus, astrocyte involvement in neuro-inflammatory events related to CSD warrants further investigation.

As detailed in Fig. 2, CSD may also produce not only a perceptual hallucination like visual aura in migraine, but may also be involved in the activation of a parenchymal cascade that leads to headache. In brief, CSD is proposed to activate headache by initiating a complex cascade where neurons open pannexin1 channels that activate caspase-1 and the release of pro-inflammatory molecules such as HMGB1 and IL-1B. Following pro-inflammatory mediator release, NF-KB translates to the nucleus to induce COX2 and iNOS expression in astrocytes. The activated astrocytes release cytokines, prosanoids, and NO to the subarachnoid space to produce sustained activation of trigeminal nerve fibers. Trigeminal fiber collaterals produce a sterile dural inflammation that lead to mast cell degranulation and the trigeminoparasympathetic reflex causes a late and sustained medial meningeal artery dilation (see Fig. S6 in Katata et al. [79]. for more details on their proposed model). In the CSD rodent model, facial grimace assessments suggest the final step in the parenchymal signaling cascade outlined above produces headache. Some notes of caution are warranted in interpreting the cascade as 1 M KCl was topically applied to the dura and may produce some of the changes observed independently of CSD. The changes observed were also following multiple CSDs elicited in a small window of time, which is not characteristic of clinical migraine. Using optogenetic and chemogenetic methods to directly target specific cellular targets in chronic models of CSD may overcome some of the difficulties in interpreting and validating the prevailing model.

Very preliminary studies using PACAP KO mice show changes in CSD (SMB, JW, unpublished observations). This is not terribly surprising given the strong vasodilatory effects of PACAP, but changes in the parenchymal intrinsic optical signal—which is a mixed signal of neural and glial activity, as well as blood volume and oxygenation, was also noted (IL, SMB, AC, JW, unpublished observations). Further work is needed to verify these results and to examine post-CSD cascades in animals with modified PACAP signaling. For example, targeting PACAP receptor sub-types and using inducible animal models to rule out compensatory mechanisms will help to delineate the role PACAP plays in CSD.

Despite the multifactorial effects of CSD on brain pathophysiology, it is important to remember that many people with migraine do not experience visual aura and some with visual aura do not report headache. This may in part be explained by where CSD is initiated, how CSD is triggered, and the extent and size of CSD changes in terms of cortical boundaries and layers. There may also be important gating mechanisms that determine whether neuro-inflammatory responses are triggered.

## Conclusion

PACAP, and activation of its receptor subtypes, clearly have a very important role in the pathophysiology of primary headache disorders. Undoubtedly this includes actions within the trigeminovascular system to activate this nociceptive pathway. However, there are also likely actions away from this medullary region, in other brain parenchyma that have direct actions related to headache pathophysiology and symptoms, as well as indirect actions involved in the modulation of dural-trigeminovascular neurotransmission. Further, the role of inflammatory mediators is likely to be intrinsic to PACAP’s mechanism of action in headache pathophysiology. In this review, we have outlined the potential role of PACAP and its receptors in neuro-inflammatory mechanisms, and how they might be related to primary headache pathophysiology. In addition, we have discussed how neuromodulatory and neuro-inflammatory mechanisms of PACAP within the brain parenchyma might also be involved in modulating dural-inflammatory mechanisms related to trigeminovascular activation and headache pathophysiology.

Our understanding of the exact role(s) of PACAP in primary headache pathophysiology is in its infancy, with much of the current research focused on its role within the trigeminovascular system, its dural innervation, and related dural inflammatory processes. There has been seminal work in dissecting some of these mechanisms, and identifying the likely PACAP receptor subtypes responsible for these actions. However, it seems that the future of this research may move away from this region and concentrate within other structures, such as the brainstem and higher nuclei, the cortex and autonomic pathways. This research is likely to concentrate on the role of PACAP in mediating associated symptoms related to primary headaches, such as migraine aura, autonomic phenomena, generalized sensory hypersensitivity and symptoms of homeostatic disturbance, as well as headache itself. With the emerging importance of PACAP in the pathophysiology of primary headache disorders, there is also ongoing development of therapeutics to target PACAP and its receptors in the context of primary headaches. This has largely followed the successful roadmap of development of CGRP-related therapeutics, with a current focus on the development of PACAP-related antibodies, targeting both PACAP and the PAC1 receptor. While preliminary data suggest that targeting PAC1 receptors might be most efficacious, we also outline arguments that targeting VPAC1 and VPAC2 receptors may still hold clinical relevance.

## Abbreviations

BBB: blood-brain barrier
CGRP: calcitonin gene-related peptide
CSD: cortical spreading depression
IV: intravenous
LC: locus coeruelus
LPS: lipopolysaccharide
NI: neurogenic inflammation
NK: natural killer
NO: nitroc oxide
NTG: nitroglycerin
PACAP: pituitary adenylyl cyclase-activating peptide
PAG: periaqueductal gray
PPE: plasma protein extravasation
RVM: rostral ventromedial medulla
SPG: sphenopalatine ganglion
SuS: superior salivatory nucleus
TACs: trigeminal autonomic cephalalgias
TCC: trigeminocervical complex
TNC: trigeminal nucleus caudalis
VIP: vasoactive intestinal peptide
